# HIGH SCHOOL CURRICULUM AND COGNITIVE FUNCTION IN THE EIGHTH DECADE OF LIFE

**DOI:** 10.1093/geroni/igae098.0275

**Published:** 2024-12-31

**Authors:** Sara Moorman, Saber Khani

**Affiliations:** Boston College, Chestnut Hill, Massachusetts, United States; Boston College, Chestnut Hill, Massachusetts, United States

## Abstract

Formal educational attainment, or years of schooling, has a well-established positive effect on cognitive health across the life course. If educators and policymakers could imbue education with even more of the vital ingredients for sustained cognitive health, then rates of cognitive impairment might continue to decline in the population as cohorts age. We hypothesized that the content and difficulty of the curriculum in high school lead to higher levels of socioeconomic attainment in adulthood and, in turn, to better cognitive outcomes in older adulthood. We estimated multilevel structural equation models (MSEMs) in data from 2,405 individuals who attended one of 1,312 U.S. high schools in 1960 and participated in the Project Talent Aging Study in 2018. Curricular difficulty (i.e., college preparatory track) and content (i.e., a greater number of semesters of math and science) in high school were positively related to cognitive function (i.e., word recall and verbal fluency) at average age 75. Effects were robust to controlling for adolescent cognitive ability, academic performance, socioeconomic background, and school characteristics. Overall educational attainment significantly mediated the association: High school curricular content and difficulty were associated with higher degree attainment, which was associated with better cognitive function in later life. We discuss implications of these findings for educational policy, where the results suggest that interventions into the content and difficulty of the high school curriculum could potentially reap cognitive benefits decades later.

